# Artificial intelligence innovations in low vision rehabilitation: a systematic review of emerging technologies

**DOI:** 10.3389/fdgth.2026.1896084

**Published:** 2026-10-15

**Authors:** Kenneth Bempah Karikari, Josephine Ampong, Sylvia Agyekum, Henry Yeboah Agyekum, Lois Manyeyo Kweiki Sabutey, Emmanuel Tettey Doku, Kwesi Sewe, Derek Kwaku Pobi Asiedu, Isaiah Osei Duah Junior, Albert Kwadjo Amoah Andoh, Werner Eisenbarth, David Ben Kumah, Kwadwo Owusu Akuffo

**Affiliations:** 1 Department of Optometry and Visual Science, College of Science, Kwame Nkrumah University of Science and Technology, Kumasi, Ghana; 2 University of Ghana Library System, University of Ghana, Legon-Accra, Ghana; 3 Department of Mathematics and Electrical Engineering, Institut Mines-Telecom (IMT) Atlantique, Brest, France; 4 Department of Psychology, John R. and Kathy R. Hairston College of Health and Human Sciences, North Carolina Agricultural and Technical State University, Greensboro, NC, United States; 5 Department of Applied Science and Mechatronics, HM Hochschule München University of Applied Sciences, Munich, Germany

**Keywords:** artificial intelligence, deep learning, low vision, low vision management, low vision rehabilitation, machine learning

## Abstract

**Background:**

Low vision is one of the major types of visual impairment worldwide and when diagnosed early can help with its management. Artificial intelligence (AI) has recently shown promising outcomes in all fields including health, with low vision rehabilitation being one of its significant applications. This systematic review aims to provide an up-to-date and comprehensive evaluation of the application of AI in the management of low vision.

**Methods:**

The systematic review followed the Preferred Reporting Items for Systematic reviews and Meta-Analyses (PRISMA) guidelines. All relevant published papers were retrieved through searches from PubMed, Scopus, Web of Science, and IEEE Xplore, without date restrictions. All retrieved articles from electronic databases were collated and managed in Covidence software.

**Results:**

Nineteen studies were included, consisting of six application domains, which include assistive navigation and wayfinding (*n* = 8), environmental interaction and object recognition (*n* = 5), reading and text access (*n* = 2), educational accessibility (*n* = 1), medical applications (*n* = 3), and digital accessibility optimization (*n* = 1). Navigation systems demonstrated the strongest performance, with leading systems achieving sub-meter location error and 100% task success rates in real-world testing. Object recognition accuracies reached 97%, hazard detection achieved 92.0% dynamic accuracy with temporal filtering, and multimodal environmental understanding received risk assessment ratings of 9.40/10. Recurring technical limitations included sensitivity to lighting conditions, processing latency, and connectivity dependency.

**Conclusion:**

AI assistive technologies for low vision demonstrate technical feasibility with promising performance. However, important evidence gaps limit clinical application: the absence of prospective validation, limited comparative effectiveness data, and minimal patient-centered outcomes. Future studies should use standardized outcomes, larger and more diverse datasets, and longitudinal evaluations to demonstrate real-world impact.

**Registration:**

https://www.crd.york.ac.uk/PROSPERO/view/CRD420261324781, CRD420261324781.

## 1 Introduction

Millions of people around the world experience low vision (1–3), a visual disability characterized by best-corrected visual acuity of less than 6/18 but equal to or better than 3/60, or a central visual field of less than 20 degrees in the better-seeing eye (4). The prevalence of chronic ocular pathologies such as age-related macular degeneration (5), diabetic retinopathy (6), glaucoma (7), and other sight-threatening conditions is rising globally (8), which is expected to substantially increase the burden of low vision (9–15). Although individuals with low vision retain some residual sight, they face significant limitations in reading (16, 17), environmental interactions (18), and spatial orientation (17), the latter of which elevates the risk of falls and related injuries (19, 20). Together, this vision loss impairs their self-reliance and autonomy in mobility (21) and increase their reliance on a surrogate person or caregiver for execution of activities of daily living (22, 23). Effective optimization of residual vision through optical and non-optical aids is therefore critical to enhance functional vision (24), support educational attainment (25), maintain occupational performance (26), and promote social engagement (27). Collectively, these considerations underscore the urgent need for evidence-based rehabilitation strategies that maximize residual visual function, facilitate independent activity, and improve overall quality of life.

Traditional low vision rehabilitation relies on optical devices, environmental modifications, and compensatory strategies to maximize residual visual function and mitigate task limitations (28). These approaches provide measurable functional benefits; however, several constraints limit their broader effectiveness and uptake. Common limitations include restricted portability, task-specific functionality, aesthetic concerns that may reduce user acceptance, and the need for prolonged training to achieve proficiency (29). In addition, most conventional aids operate as static tools without the capacity to respond to dynamic environmental changes or evolving user requirements. Economic barriers further restrict access, particularly in low-resource settings, where availability and affordability remain persistent challenges (30).

Recent advances in artificial intelligence (AI), particularly in machine learning and computer vision, have introduced a fundamentally different approach to assistive technology (31–35). These systems enable real-time analysis and interpretation of visual environments, which allows active and context-aware support for users with low vision (31–35). Deep learning models can perform complex tasks such as object detection, text recognition in natural scenes, facial identification, and scene interpretation with high accuracy (36, 37). Computer vision frameworks based on convolutional neural networks (CNN) support a wide range of functional applications, including reading handwritten or printed text, identifying currency and everyday objects, detecting obstacles, and interpreting social and environmental cues (38). Such capabilities extend beyond static compensation and offer adaptive assistance that aligns with real-world task demands (39).

Existing reviews have broadly examined AI technologies for visual impairment and blindness (40–44, 46) but have not specifically focused on low vision rehabilitation. While they provide insights into specific device categories such as smart canes and wearable systems (41, 43, 45), and have successfully mapped technical metrics (41, 47), they often focus on a sensory-substitution paradigm that seeks to replace sight entirely, rather than supporting residual vision. Methodologically, previous reviews have failed to adequately investigate real-world clinical outcomes, focusing instead on laboratory performance while overlooking how these interventions promote independence and task performance in daily living (44, 47). Additionally, critical implementation barriers such as high energy consumption (41, 44, 48), prohibitive costs (45), and a lack of digital accessibility (49) remain under-explored, leaving a gap in our understanding of how these technologies can be scaled equitably. Addressing these unmet needs is essential, as the rapid pace of innovation quickly renders existing evidence syntheses outdated and a comprehensive evaluation of effectiveness, usability, and equity is necessary to translate AI prototypes into practical tools that meaningfully enhance autonomy for individuals with low vision.

This systematic review addresses this knowledge gap through a comprehensive and up-to-date synthesis of evidence on AI applications in low vision rehabilitation. Specifically, the review aimed to classify these technologies based on functional domains, including navigation, reading, and object recognition, and to evaluate outcomes related to visual function, task performance, and independence. In addition, this review identifies technical limitations in current research and technology development. By consolidating the available evidence, this work provides a structured foundation to inform clinical practice, guide future research, and support technology development. Taken together, these findings will offer practical value for clinicians in technology selection, for researchers in setting priorities for further investigation, for developers in aligning innovations with user needs, and for policymakers in resource allocation. Importantly, this review also supports individuals with low vision and their families in making informed decisions regarding assistive technology options.

## 2 Methods

This systematic review followed the PRISMA guidelines (50). The protocol was registered with the International Prospective Register of Systematic Reviews (PROSPERO) (ID: CRD420261324781) prior to commencing the full systematic review. PRISMA flow diagram was used to report the study selection process (51).

### 2.1 Inclusion criteria

#### 2.1.1 Population

Individuals of any age with low vision, including both congenital and acquired causes. Studies involving normally sighted participants were included only where they evaluated low vision–specific technologies in simulated low-vision settings.

#### 2.1.2 Technologies

Digital technologies and AI systems designed to assist individuals with low vision in rehabilitation or daily functioning, including but not limited to mobile applications, tablet-based systems, computer software, electronic magnification devices, wearable technologies, smart glasses, virtual and augmented reality systems, telemedicine platforms, and smart home technologies; and AI technologies including machine learning and deep learning models, computer vision (object, face, and text recognition/OCR), scene understanding and description, natural language processing, predictive analytics, and adaptive personalization systems. Both commercially available products and technologies under development in research settings were included.

#### 2.1.3 Comparators

Standard low vision rehabilitation approaches, other digital technologies, or no comparator, depending on study design; studies without a comparator were eligible.

#### 2.1.4 Study designs

Peer-reviewed empirical studies of any design, including randomized controlled trials, quasi-experimental studies, cohort studies, cross-sectional studies, case series, usability studies, prototype evaluations, and mixed-methods research.

#### 2.2 Exclusion criteria

Studies focused exclusively on individuals with complete blindness, without findings applicable to people with low vision.
Studies addressing only the prevention, diagnosis, or medical/surgical treatment of eye diseases, without a vision rehabilitation component.
Publications that did not present original primary research, including systematic reviews, literature reviews, scoping reviews, conference abstracts, editorials, commentaries, expert opinions, perspectives, case reports, study protocols, and unpublished reports.
Studies with insufficient methodological detail, or not available in full text.

### 2.3 Information sources and search strategy

Comprehensive searches were conducted in multidisciplinary, biomedical, and engineering databases to capture both clinical and technical literature. The following databases were searched from inception to 27th February 2026: PubMed, Scopus, Web of Science, and IEEE Xplore. Search strategies were tailored to each database using a combination of controlled vocabulary and free-text terms related to low vision, AI technologies, and low vision rehabilitation. Boolean operators were applied as appropriate. A detailed search strategy was developed and peer-reviewed by an information specialist for all databases (Supplementary file 1).

### 2.4 Study selection

All retrieved records were imported into Covidence systematic review software (52) for screening and data management. Two reviewers independently screened all titles and abstracts using a predetermined eligibility criteria. Full-text articles were obtained for all records that passed the title and abstract screening. Two reviewers independently assessed each full-text article against all eligibility criteria using a standardized full-text screening form that documented reasons for exclusion. Disagreements at both screening stages were resolved through discussion between the two reviewers, with a third reviewer consulted when consensus could not be reached.

### 2.5 Data extraction

A standardized data extraction form was developed and piloted on five diverse studies representing different study designs, technologies, and populations. Based on the pilot testing, the form was refined to ensure clarity, completeness, and feasibility. The final form captured all relevant data needed to address the review objectives. Extracted items included study characteristics including, author, year, purpose of study, AI task, AI model, AI application domain, input data, output data, platform and performance metrics, and key limitations. Two reviewers independently extracted data, and discrepancies were resolved by consensus.

### 2.6 Data synthesis

Due to heterogeneity in study designs, interventions, outcomes, and evaluation methodologies, the findings were synthesized narratively.

## 3 Results

A comprehensive search across the selected databases identified 2,789 records. After removing 369 duplicates, 2,420 records were screened. Of these, 2,386 were excluded, leaving 34 articles for full-text assessment. A further 15 studies were excluded with reasons provided (Figure 1). Ultimately, 19 studies met the inclusion criteria and were included in the qualitative synthesis. Figure 1 shows the PRISMA flow diagram for the screening process.

**Figure 1:**
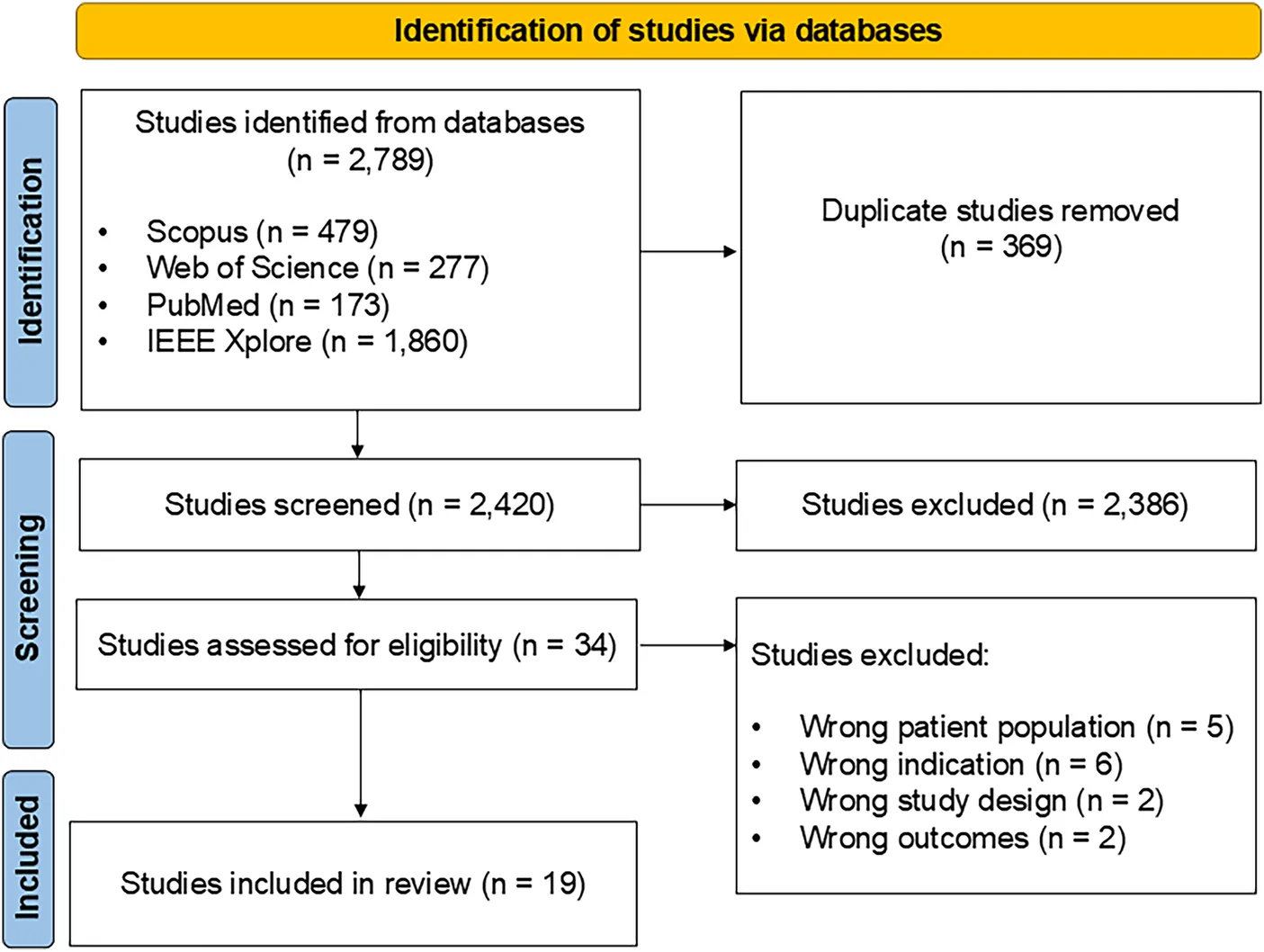
PRISMA flow diagram.

### 3.1 Study characteristics

This systematic review identified 19 studies published between 2015 and 2026 investigating AI applications in low vision rehabilitation (Table 1). Publication activity increased notably in recent years, with the majority of studies published between 2022 and 2026.

**Table 1:** Study characteristics**.**

Author, Year	Purpose	AI Application	AI Task	AI/Model	Input Data Type	Output/Feedback	Platform
Chen et al. 2025 (53)	To develop a wearable walking-assistance device	Outdoor navigation Walking assistance	Instance segmentation	YOLOv8n-seg	Real-time camera images	Tactile feedback conveying environmental information to the user	NVIDIA Jetson Nano
Croce et al. 2019 (54)	To validate a smartphone-based navigation system	Assistive navigation	Path recognition; landmark detection; user pose estimation	Computer vision with Extended Kalman Filter	Images; sensor data	Tactile vibration signals and audible information via landmarks	Smartphone
Dai et al. 2020 (35)	To predict appropriate assistive devices for low vision patients using AI	Assistive device fitting	Multi-label classification	Fully connected neural network	Visual function, rehabilitation needs, and quality of life scores	Predicted classification of suitable assistive devices	Clinical AI model
Dai et al. 2025 (33)	To develop an AI-powered model for automated LVA fitting	Assistive device fitting	Multi-category binary classification to predict prescriptions for three LVA types	Random Forest Deep Neural Network Logistic Regression	Clinical and demographic data	Data-driven prescription recommendations	Cloud server
Feng et al. 2023 (55)	To offer trip planning and guidance inside subway transit hubs	Assistive indoor wayfinding	OCR-based sign detection and navigation guidance	Google ML Kit	Real-time video frames	Haptic vibration and verbal navigation instructions (TTS)	Smart devices
Feng et al. 2026 (56)	To develop and evaluate a system for construction hazard detection for safer mobility	Hazard identification and mobility support	Open-vocabulary object detection and construction hazard recognition	YOLO-World, YOLOv8, and PaddleOCR 3.0	Video footage	Real-time audio alerts	NVIDIA Jetson Orin NX
Hamilton-Fletcher et al. 2024 (57)	To estimate distances using smartphone-based AI	Assistive smartphone application	Monocular depth estimation	Machine Learning (ResNet-50)	RGB images, IR grid distortion, LiDAR pulses	Visual depth maps indicating proximity	Apple iPhones
Hao et al. 2024 (58)	To assist with scene understanding and hazard detection	Environmental interaction and navigation	Scene understanding; object localization; risk assessment	Multimodal foundation models (RAM, InstructBLIP, Vicuna-13B)	Camera images; voice queries	Audio scene descriptions, object localization, and risk assessment alerts	Smartphone + cloud server
Huang et al. 2019 (59)	To support sign reading and indoor wayfinding	Indoor wayfinding and information access	Optical character recognition; text localization; text-to-speech	Computer Vision	HMD camera images	AR visual cues (flashing indicators/magnified text) and text-to-speech	Microsoft HoloLens
Li et al. 2024 (60)	To improve image recognition for visual prosthesis systems	Visual prosthesis support	Image recognition and classification	Optimized VGGNet-19 with a Fruit Fly Optimization Algorithm	Low-resolution RGB images	Encoded electrical signals transmitted to the optic nerve to create artificial vision	Prosthesis image processor
Park et al. 2026 (61)	To detect visual elements in videos and generate natural language descriptions	Educational accessibility	Chart detection, tracking, and natural language captioning	YOLOv8	Lecture video frames	Spoken and textual descriptions; automatic video pausing	Web-based cloud platform
Sankarnarayanan et al. 2023 (62)	To train AI to recognize objects specifically relevant to pBLV	Daily functioning assistance	Object localization and classification	YOLOv5x	RGB images	Bounding boxes with class labels and confidence scores	Smartphones
Sin et al. 2015 (63)	To develop a mobile text reader with image stitching	Assistive reading	Image stitching; Optical Character Recognition; Text-to-Speech	Harris corner detection with RANSAC and ABBYY OCR	Smartphone camera images	Natural-sounding speech and visual display of recognized text	Windows Phone
Sofiyati et al. 2024 (64)	To optimize smartphone-based image classification	Mobility and object recognition	Image classification (indoor, outdoor, currency)	CNN (VGG16-based)	Camera frames	Guidance voice identification of objects and visual accuracy percentages	Android smartphone
Sun et al. 2025 (65)	To compare scene-specific and generic segmentation models	Indoor scene understanding	Semantic segmentation	DeepLabV3+	RGB images	Multimodal feedback including verbal descriptions, spatialized tones, or vibrotactile alerts	Smartphone-compatible
Yang et al. 2022 (66)	To develop infrastructure-free visual localization	Autonomous navigation	Visual place recognition; image-based localization	Integrated Deep Neural Networks (NetVLAD); SuperPoint, SuperGlue	360° video; RGB images	Auditory or vibration navigation instructions via a bone-conduction headset	Android + wearable system
Yang et al. 2025 (67)	To evaluate the UNav navigation system in unfamiliar environments	Vision substitution navigation aid	Visual place recognition and localization	Deep learning pipeline (DGX A100 infrastructure)	Query images	Audio step-by-step navigation guidance	Wearable backpack + remote server
Zhang et al. 2022 (68)	To segment transparent objects and semantic scenes	Wearable assistive navigation	Semantic segmentation of transparent and general objects	Trans4Trans (Dual-head Transformer)	RGB and depth images	Acoustic alerts and vibration feedback	Smart vision glasses + mobile GPU
Zhang et al. 2024 (69)	To automatically fix GUI accessibility issues	Digital accessibility	GUI graph learning and optimization	R-GCN	GUI layout data	Concrete attribute adjustments (size, color, interval) for code updates	Android apps

AI, artificial intelligence; AR, augmented reality; CNN, convolutional neural network; GUI, graphical user interface; GPU graphics processing unit; HMD, head-mounted display; IR infrared; LVA, low vision aid; OCR, optical character recognition; pBLV, people with blindness and low vision; RANSAC, random sample consensus; R-GCN, relational-graph convolutional neural network; RGB red, green and blue; TTS, text-to-speech.

### 3.2 AI application domains

Six primary application domains emerged from the review based on each study's common theme and primary technical function. Assistive navigation and wayfinding were the most common applications (*n* = 8) (53–56, 59, 66–68), addressing indoor and outdoor mobility through path recognition, landmark detection, pose estimation, visual place recognition, construction hazard detection and transparent object segmentation. Environmental interaction and object recognition accounted for five studies (57, 58, 62, 64, 65), followed by reading and text access (*n* = 2) with one study exhibiting both navigation and reading properties (59, 63). An emerging domain of educational accessibility was identified (*n* = 1) (61), addressing access to visual content in lecture environments. Specialized applications included digital accessibility optimization (*n* = 1) (69) and medical applications including visual prosthesis support (60) and clinical decision support (33, 35) (Table 2, Figure 2).

**Table 2:** Summary of performance metrics by AI application.

AI application	Study	Primary metrics	Best performance	Key limitations
Navigation & wayfinding	Croce et al. (54)	Mean heading error	2.35° ± 7.39°^2^	Lighting sensitivity; drift during turns
	Yang et al. (66)	Location error	<1 meter average	Processing latency; reference density dependency
	Huang et al. (59)	Route efficiency; Task time	Distance ratio: 0.36 vs. 0.50 control; +129s completion time	3–4s OCR latency; WiFi dependency
	Chen et al. (53)	Segmentation accuracy	Precision: 0.905; F1: 0.912; mIoU: 85.85%	Occlusion; missing curbstones not reported
	Feng et al. (55)	Detection accuracy, detection range, and field of view angle	0.97 accuracy; 11 m maximum detection range; 110-degree FOV	Struggles with purely graphical signs; reliance on unfinished GTFS pathway datasets
	Yang et al. (67)	Navigation success, path efficiency, wrong turns	100% success rate; better path efficiency (M = 1.17, *p* < 0.001); fewer wrong turns (M = 0.049, *p* < 0.001)	High round-trip latency (3.62s); dependency on network/server connectivity
	Zhang et al. (68)	mIoU accuracy, system latency, and cognitive load	mIoU=75.1%; Accuracy=95.01%; Cognitive load=16.3	2-second feedback cycles caused delay inconsistencies; background textures interfere with glass segmentation
	Feng et al. (56)	Accuracy, angular offset, detection distance, and event-based alarm rate	88.56% overall static accuracy; 92.0% dynamic accuracy with temporal filtering	Motion blur during sharp turns; viewpoint dependency (e.g., barricades); camera lacks autofocus
Environmental interaction & object recognition	Hao et al. (58)	User ratings (1–10 scale)	Scene understanding: 8.85; Object localization: 8.60; Risk assessment: 9.40	Lighting/weather sensitivity; false alarms
	Sofiyati et al. (64)	Classification accuracy	Indoor: 95.5%; Outdoor: 96.79%; Currency: 93.35%	Quantization accuracy loss
	Sun et al. (65)	Mean accuracy; mIoU	Kitchen: 43.11% (0.234); Bedroom: 34.65% (0.275); Bathroom: 0.363 mIoU	Extreme low-light degradation
	Hamilton-Fletcher et al. (57)	Central/peripheral distance error, CPU usage, battery usage, field-of-view (FoV)	CPU=44%; FoV=52 degrees	ML less accurate than sensors; high battery drains with LiDAR/face-tracking; accuracy drops in periphery
	Sankarnarayanan et al. (62)	Mean average precision (mAP50–95)	Original mAP50-95 = 0.199; Curated mAP50-95 = 0.413	Performance in cluttered environments uncertain; ability to recognize absence of objects unknown
Reading & text access	Sin et al. (63)	Processing time	31–66 s for image stitching	WiFi dependency; camera alignment
	Huang et al. (59)	OCR processing	3–4 s per sign	Cloud processing dependency, time cost
Medical Applications	Li et al. (60)	Recognition accuracy; AUC	68.29–97.23%; AUC: 0.942; F1: 91.47%	Limited efficiency optimization
	Dai et al. (35)	Multi-label classification	Accuracy: 0.65; Precision: 0.4; Recall: 0.9; F1:0.31 at low threshold of 0.1	Low precision; no prospective validation
	Dai et al. (33)	AUC, ACC, SEN, SPE, and F1 score	DOV: ACC=0.78, SEN=0.61, SPE = 0.98, F1 = 0.75, AUC=0.93 NEV: ACC=0.79, SEN=0.50, SPE=0.99, F1 = 0.66, AUC=0.83 NOV: ACC=0.87, SEN=0.83, SPE=0.90, F1 = 0.84, AUC=0.91	Single ethnicity (Chinese cohort); lack of longitudinal data on device abandonment; and potential overfitting
Educational accessibility	Park et al. (61)	Precision, Recall, F1-Score, BLEU-4	Precision=89.1%; Recall=83.3%; F1-Score=86.1%; BLEU-4 = 36.7	Semantic hallucinations; lacks support for 3D or composite charts
Digital accessibility	Zhang et al. (69)	Issue resolution rate	81.2% automatic fixes; 84.5% user-reported problem reduction	Cannot fix image-embedded issues

ACC, accuracy; AUC, area under the curve; DOV, distant optical visual aids; GTFS, general transit feed specification; mIoU, mean intersection over union; NEV, near electronic visual aids; NOV, near optical visual aids; OCR, optical character recognition; pBLV, people with blindness and low vision; SEN, sensitivity; SPE, specificity; TTS, text-to-speech.

**Figure 2:**
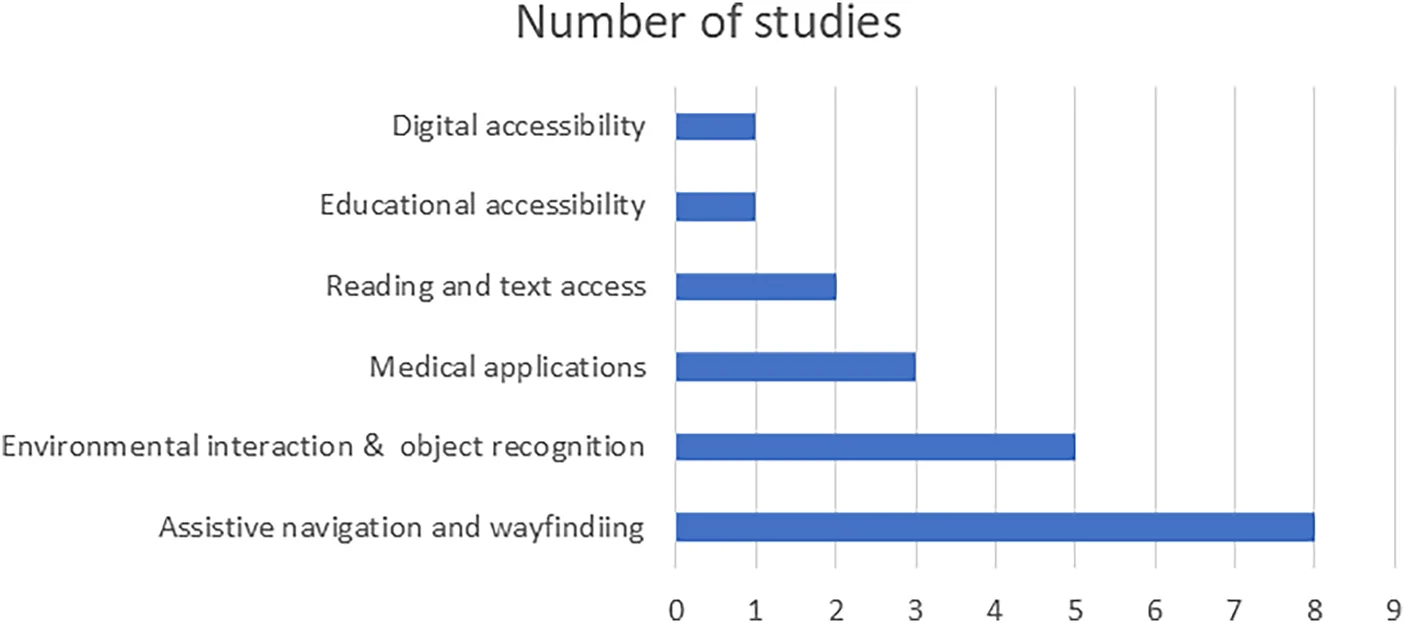
Distribution of studies by AI application domain.

### 3.3 Performance outcomes

Performance evaluation across the 19 included studies varied considerably in testing context and outcome type. Four studies (21%) reported performance under real-world field conditions involving deployment beyond controlled testing environments: the extended evaluation of UNav in unfamiliar real-world environments (67), the construction hazard detection system tested in live outdoor construction zones (56), the multimodal foundation model evaluated through user ratings during real-world scene interaction (58), and the digital accessibility repair system evaluated via real-world application use (69). The remaining studies reported performance derived from offline, dataset- or bench-based testing, with accuracy, F1-score, mIoU, or AUC computed on held-out test data rather than through live deployment. Dataset sizes were inconsistently reported across studies, and few explicitly confirmed evaluation on an independent dataset distinct from training or development data. This section organizes the included studies by technical function and outcome metrics (e.g., navigation, classification, segmentation and processing speed).

#### 3.3.1 Navigation systems

Navigation systems demonstrated varying levels of localization accuracy depending on technical approach and environmental complexity. The infrastructure-independent visual navigation system (UNav) achieved the most precise performance with average location errors below 1 meter across tested environments (dataset-based testing) (66). The extended evaluation of UNav in unfamiliar environments (67) confirmed robust real-world deployment, achieving a 100% navigation success rate with significantly better path efficiency (M = 1.17, *p* < 0.001) and fewer wrong turns (M = 0.049, *p* < 0.001) compared to unassisted navigation representing one of only four included studies to report a patient-facing functional outcome. However, the system incurred high round-trip latency of 3.62 s and remained dependent on network and server connectivity, which affected responsiveness in dynamic navigation scenarios.

The sensor fusion approach combining computer vision with Extended Kalman Filter reported mean heading error of 2.35° with 7.39°^2^ variance (54). The wearable curbstone detection system achieved high instance segmentation performance with precision of 0.905, recall of 0.928, F1-score of 0.912, and mean Intersection-over-Union (mIoU) of 85.85% (dataset-based testing) (53). The subway transit wayfinding system (55) demonstrated strong sign detection performance within complex transit environments, achieving 0.97 detection accuracy with a maximum detection range of 11 meters and a 110-degree field of view using Google ML Kit for OCR-based sign detection, with haptic vibration and TTS feedback supporting route following. Performance was limited by reliance on purely graphical signs lacking text and dependency on incomplete GTFS pathway datasets.

Zhang et al. (68) demonstrated that the dual-head Trans4Trans transformer model achieved mIoU of 75.1% and overall accuracy of 95.01% across transparent and general object segmentation tasks in wearable assistive navigation(dataset-based testing). However, 2-second feedback cycles introduced delay inconsistencies, and background textures were found to interfere with glass segmentation, limiting reliability in visually complex environments. Route efficiency improvements were demonstrated in the augmented reality sign-reading system, where users achieved shorter, more direct navigation paths (mean distance ratio 0.36 vs. 0.50 in the control condition, representing a 28% improvement) in a live user study, one of the four included studies to report a patient-facing functional outcome (59). However, this accuracy improvement came with a significant time trade-off: AR users required approximately 129 s longer to complete navigation tasks and walked at slower speeds than during unassisted navigation. This performance pattern revealed a fundamental tension between route optimization and task completion efficiency.

#### 3.3.2 Image classification and object recognition performance

Image classification systems achieved robust accuracy across well-defined recognition tasks. The multi-category classification system (LoVi App) demonstrated strong performance with testing accuracy of 95.5% for indoor environment classification, 96.79% for outdoor/sidewalk classification, and 93.35% for currency recognition (dataset-based testing) (64).

The visual prosthesis image recognition system showed substantial performance variability across object categories, with accuracy ranging from 68.29% to 97.23% (the highest single-task accuracy) depending on task complexity and visual characteristics(dataset-based testing) (60). Overall discriminative capability was strong (AUC=0.942) with balanced precision-recall performance (maximum F1-score=91.47%). This wide performance range reflected differential difficulty across recognition contexts, with simple high-contrast objects achieving near-perfect recognition while complex or low-contrast items remained challenging for low-resolution prosthetic vision input.

#### 3.3.3 Depth estimation performance

Hamilton-Fletcher et al. (57) evaluated monocular depth estimation using a ResNet-50 model on Apple iPhones, fusing RGB images, infrared grid distortion, and LiDAR pulses to generate visual depth maps for proximity estimation(dataset-based testing). The system achieved a CPU utilization of 44% and a functional field of view of 52°. Machine learning-based estimation was less accurate than raw sensor readings, and LiDAR combined with face-tracking features imposed high battery drain. Accuracy dropped notably in the peripheral field of view, limiting utility for detecting hazards at the edges of the user's visual field.

Sankarnarayanan et al. (62) trained YOLOv5x on a curated dataset of objects specifically relevant to people with blindness and low vision, improving mAP50-95 from 0.199 on the original dataset to 0.413 on the curated dataset which is a 107% relative improvement, demonstrating the substantial benefit of domain-specific data curation over general object detector fine-tuning. Evaluation was conducted on dataset-based testing, and independent-dataset validation was not reported. Performance in cluttered environments remained uncertain, and the model's capacity to recognize the absence of expected objects was not evaluated.

#### 3.3.4 Semantic segmentation performance

Semantic segmentation models for indoor scene recognition showed moderate performance and provided valuable insights about specialization strategies. Scene-specific models outperformed generic models across most evaluated environments. Kitchen-specific models achieved 43.11% mean accuracy compared to 29.10% for generic models (48% relative improvement), with mIoU improving from 0.210 to 0.234 (11% relative gain) (65). Bedroom segmentation achieved 34.65% scene-specific accuracy, vs. 30.77% generic performance (13% relative improvement), and increased mIoU from 0.229 to 0.275 (20% relative gain). Bathroom environments demonstrated the largest performance differential, with scene-specific models achieving mIoU of 0.363 compared to 0.230 for generic models (58% relative improvement).

Notably, living room segmentation presented an exception to this pattern, with generic models achieving 33.85% mean accuracy compared to 31.16% for scene-specific models, and mIoU of 0.269 vs. 0.244 respectively. This reversed performance suggested that living rooms exhibit greater spatial and functional heterogeneity that benefits from broader training data rather than environment-specific optimization (65). Zhang et al. (68) addressed the distinct challenge of transparent object segmentation using a dual-head transformer (Trans4Trans), demonstrating that a dedicated segmentation head for transparent surfaces improved performance over unified models that conflate transparent and opaque objects, a practically important distinction for wearable navigation where glass doors, windows, and reflective surfaces pose hazard risks.

#### 3.3.5 Environmental understanding and multimodal interaction

The multimodal foundation model for environmental interaction achieved high user ratings across three functional domains on a 10-point rating scale (58). Scene understanding scored 8.85/10, indicating strong capability in generating comprehensive environmental descriptions including object identities, spatial relationships, and contextual interpretation. Object localization performance of 8.60/10 demonstrated effective spatial referencing enabling users to locate objects within their environment. Risk assessment achieved the highest rating of 9.40/10, indicating strong system performance in identifying potential hazards, including obstacles, drop-offs, and unstable surfaces.

The construction hazard detection system (56), deploying YOLO-World, YOLOv8, and PaddleOCR 3.0 on an NVIDIA Jetson Orin NX, demonstrated strong performance in identifying construction-zone hazards in real-time outdoor environments. The system achieved 88.56% overall static accuracy, improving to 92.0% dynamic accuracy with temporal filtering applied across video frames. Key limitations included motion blur during sharp turns, viewpoint-dependent failures for objects such as barricades viewed at oblique angles, and the absence of autofocus in the camera module, which reduced detection reliability for distant or small hazard objects.

#### 3.3.6 Educational accessibility

Park et al. (61) applied YOLOv8 on a web-based cloud platform to detect and track charts in lecture videos, generating spoken and textual captions and automatically pausing video playback to allow processing time. The system achieved precision of 89.1%, recall of 83.3%, and F1-score of 86.1% for chart detection, with a BLEU-4 score of 36.7 for caption generation quality. Key limitations included semantic hallucinations in generated captions and a lack of support for 3D or composite chart types, restricting coverage of the full range of visual content encountered in academic settings.

#### 3.3.7 Processing efficiency and real-time performance

Processing latency emerged as a significant constraint across multiple application domains. Text reading applications demonstrated the most substantial processing delays, with image stitching requiring 66 s for two 1024 × 576- resolution images or 31 s for five 844 × 475-resolution images (63). This inverse relationship between image count and processing time across resolutions demonstrated that resolution reduction yielded greater computational savings than the penalties of increased image count, though both processing times remained impractical for time-sensitive reading tasks.

OCR processing introduced a 3 to 4 s latency per sign in the augmented reality navigation system (59). Edge-deployed systems [NVIDIA Jetson Nano (53); NVIDIA Jetson Orin NX (56)] achieved real-time inference for object detection and segmentation tasks without cloud dependency, representing an important architectural advance for latency-sensitive navigation applications. The UNav server-dependent pipeline (67) incurred variable latency associated with remote processing, which affected responsiveness in dynamic navigation scenarios. Model quantization for mobile deployment introduced accuracy degradation as a trade-off for reduced computational requirements (64), and the visual prosthesis study acknowledged insufficient attention to processing efficiency despite strict power and thermal constraints in wearable systems (60).

#### 3.3.8 Clinical decision support performance

The multi-label classification system for assistive device prediction demonstrated an overall modest performance with notable precision-recall imbalance (35). Accuracy of 0.65 indicated that approximately two-thirds of device recommendations were correct. Precision of 0.4 indicated that only 40% of positive device recommendations were actually appropriate, suggesting substantial false-positive rates. Recall of 0.9 demonstrated that the model successfully identified 90% of truly suitable devices. The F1 score of 0.31 highlighted a severe imbalance between these complementary metrics, suggesting a liberal recommendation strategy that over-predicted device suitability to minimize missed opportunities at the cost of many inappropriate recommendations.

Building on their earlier work, Dai et al. (33) developed a more refined AI-powered model for automated low vision aid fitting, applying Random Forest, Deep Neural Network, and Logistic Regression models to clinical and demographic data across three device categories. Performance varied by device type: distance optical visual aids (DOV) achieved the highest discriminative capability (AUC=0.93) with strong specificity (0.98) but moderate sensitivity (0.61); near electronic visual aids (NEV) demonstrated acceptable discrimination (AUC=0.83) with similarly high specificity (0.99) but lower sensitivity (0.50); and near optical visual aids (NOV) achieved the most balanced performance overall, with accuracy of 0.87, sensitivity of 0.83, specificity of 0.90, F1-score of 0.84, and AUC of 0.91. Across all three categories, specificity consistently exceeded sensitivity, suggesting the model was conservative in recommending devices, prioritizing the avoidance of inappropriate prescriptions over comprehensive identification of suitable aids.

#### 3.3.9 Digital accessibility optimization

The graph convolutional network for automated GUI accessibility repair resolved an average of 81.2% of identified accessibility issues across tested applications, demonstrating a high success rate in automatically implementing fixes for common violations including insufficient color contrast, inadequate touch target sizes, and problematic spacing (69). User evaluation demonstrated 84.5% reduction in accessibility problems identified during real-world application use. The system achieved a 3.54% higher repair rate for size-related accessibility issues than baseline automated tools.

### 3.4 Technical limitations in included studies

#### 3.4.1 Environmental and operational constraints

Technical limitations constrained system reliability and deployment feasibility across diverse real-world contexts. Lighting variations impaired performance in five studies (26%) (57, 58, 62, 64, 65). Systems demonstrated reduced accuracy under low light conditions (evening hours, poorly lit interiors), high-contrast illumination (harsh shadows, backlighting), and rapidly changing lighting (moving from indoors to outdoors, cloud cover variations). These sensitivities proved particularly problematic since low-vision users may require assistive support most urgently during challenging visual conditions when residual vision provides minimal useful information.

Adverse weather degraded performance by affecting image quality and visibility of environmental feature (58). Rain, fog, and snow reduced image contrast and introduced visual noise. Crowded environments created uncertain performances (62). Visual clutter in complex scenes challenged object detection and sign localization algorithms (59).

Connectivity requirements limited deployment flexibility, with a reliance on WiFi restricting use to network-covered areas and excluding outdoor environments and public buildings without guest access (59, 63). Cloud-based processing, while enabling sophisticated models that exceed mobile computational capabilities, introduced latency and increased vulnerability to network interruptions. Camera alignment requirements demanded precise positioning, which is difficult for low-vision users who cannot visually verify image framing (63).

#### 3.4.2 Processing and computational constraints

Real-time performance limitations affected four studies (21%). OCR processing delays of 3–4 s per sign disrupted natural navigation flow (59). Image stitching times of 31–66 s rendered systems impractical for time-sensitive reading tasks (63). General system latency in visual place recognition affected real-time responsiveness (66). The visual prosthesis study acknowledged insufficient attention to processing efficiency despite strict power and thermal constraints in wearable/implantable systems (60).

Navigation systems encountered multiple challenges in spatial accuracy (53, 54, 66–68). Reduced accuracy during sharp turns suggested gyroscope drift or visual tracking failure during rapid orientation changes. Dead-reckoning drift accumulated over extended use periods, particularly in GPS-denied indoor environments. Three-dimensional localization offsets in augmented reality applications caused spatial misalignment between virtual information and physical referents, creating confusion or navigation errors. Dependence on reference frame density created performance variability based on the completeness of the environment documentation in the UNav systems (66, 67).

Of the included studies, 4 incorporated a user-facing functional or usability outcome: the extended evaluation of UNav, which assessed navigation success rate; the augmented reality sign-reading system, which measured route efficiency and task completion time in a live user study; the multimodal foundation model for environmental interaction, which used 10-point user ratings of scene understanding; and the digital accessibility repair system, which reported a real-world reduction in user-encountered accessibility problems. No included study administered a formal, validated quality-of-life or functional vision instrument. One study assessed the cognitive load of wearers but cautioned the findings reflect experts' evaluation task rather than real-world user workload (53). The remaining 14 studies reported exclusively technical performance metrics without a corresponding patient-reported or functional outcome measure.

## 4 Discussion

### 4.1 Summary of key findings

Due to the increasing prevalence of low vision worldwide and the growing sophistication of AI technologies, a substantial body of research has emerged investigating the utilization of AI in assistive applications for low vision rehabilitation. The majority of works in the literature employ smartphone-based platforms, whereas a minority use specialized hardware including head-mounted displays, wearable devices, and embedded systems. It is worth noting that navigation applications are the main focus of studies that perform real-time environmental processing, with CNNs as the dominant architecture for its real-time navigation due to their lower inference latency on mobile platforms; conversely, studies that focus on environmental interaction primarily use multimodal foundation models and semantic segmentation.

Across navigation and localization studies, performance was generally strong and clinically useful, with sub-meter localization accuracy (≤1 m) (66), low mean heading error (2.35°) (54), and effective detection of landmarks and curbstones for outdoor mobility (53, 54). While most systems relied on RGB images, the ability of using 360° video or visual-inertial sensor fusion reported more stable localization and orientation estimates than vision-only setups. Multi-task systems also showed strong performance across functions, including effective OCR for sign reading (59, 63), accurate environmental hazard detection and risk assessment (58), and high classification accuracy for indoor/outdoor scenes and currency recognition (≥93%–95%) (64). All these validate the potential and necessity of AI-powered applications and services to support individuals with low vision in daily life, echoing similar conclusions from previous studies (31, 70).

Based on the research analyzed in this review, AI systems achieved high accuracy in well-defined tasks. Classification accuracy exceeded 95% for indoor and outdoor scenes and 93% for currency recognition (64). Visual prosthesis recognition performance varied by object complexity (68.29–97.23%), but overall discriminative ability remained strong (AUC=0.942; F1 = 91.47%) (60). Multimodal foundation models achieved high user-rated performance for scene understanding, object localization, and hazard detection (8.60–9.40/10) (58), supporting their potential for safe mobility. These findings are also supported by previous research emphasizing the unique needs of people with low vision, highlighting further opportunities to improve low vision accessibility tools (71, 72). However, these applications should be developed and formally evaluated, which remains a primary recommendation for future work in this field.

Furthermore, the diversity of AI architectures employed across studies led to the substantial variation in technical approach and application domain. While traditional computer vision with sensor fusion demonstrated robust localization for navigation (54), integrated deep learning pipelines achieved the highest spatial accuracy with sub-meter location errors (66). Overall, AI performance was strongly context-dependent, indicating the importance of environment-aware model design.

In the following discussion, we identify three recurring constraints that limit translation to everyday use: sensitivity to real-world environmental variation, computational demands incompatible with continuous mobile deployment, and inconsistent evaluation practices that limit comparability across studies.

### 4.2 Areas for enhancing current AI assistive technologies

#### 4.2.1 Robustness to real-world environmental conditions

Several reviewed systems were evaluated under relatively stable conditions, and performance variability was most pronounced where lighting, weather, or occlusion diverged from training conditions as visual prosthesis recognition accuracy ranged from 68.29% to 97.23% depending on object complexity (60). To reduce the effects of lighting variations, both hardware and software design aspects must be addressed, integrating adaptive exposure control systems that automatically adjust camera settings based on ambient lighting conditions is crucial, which is similar to ëxposure used in HDR imaging (73, 74). Some devices lack dynamic exposure adjustment, leading to over-or underexposed images that degrade recognition accuracy. Additionally, incorporating multi-spectral imaging sensors that capture information beyond the visible light spectrum, such as near-infrared, enables systems to maintain performance in challenging lighting conditions (75–77). In software designs, image preprocessing techniques will be essential for normalizing lighting variations prior to model inference (78). Systems relying solely on RGB input were also more prone to degraded localization than those using sensor fusion, which produced more stable estimates.

To decrease the effect of these spatial errors on navigation safety and user confidence, both sensor fusion and error correction aspects must be addressed. In sensor fusion design, implementing multi-modal approaches that combine visual odometry, inertial measurement units (IMUs), magnetometers, and GPS/GNSS as some systems rely on a single sensing modality, leading to error accumulation over extended use or in GPS-denied environments (89). On the error correction side, algorithmic techniques are essential for maintaining long-term accuracy. For instance, loop closure detection algorithms recognize previously visited locations and correct accumulated drift through global pose graph optimization, enabling accurate long-term navigation (90).

Two directions are most directly supported by these findings. First, multi-modal sensor fusion which outperforms vision-only setups for localization (54) should be extended to recognition and hazard-detection tasks, combining visual data with depth, infrared, or inertial sensing to reduce reliance on any single, lighting- or weather-sensitive modality. Second, training on degraded or domain-shifted imagery than standard high-quality computer vision datasets would directly address the accuracy variability observed in prosthetic vision recognition (60).

This variability, observed in a single system, highlights generalization challenges when AI models encounter low-resolution and low-contrast conditions. In practice, this would involve creating specialized datasets capturing the low-resolution, low-contrast conditions characteristic of prosthetic vision. Curriculum learning strategies, which train models on increasingly difficult examples, may also systematically build robustness to challenging recognition scenarios (91).

#### 4.2.2 Computational efficiency and deployment constraints

Different types of AI models generate varying levels of computational demand. This can be addressed by implementing neural architecture search (NAS) to discover efficient model architectures optimized for target hardware platforms while maintaining accuracy (79). Dynamic neural networks with early-exit mechanisms adjust computational complexity based on input difficulty, processing simple cases faster while allocating more computation to challenging inputs (80).

Model compression (pruning, quantization, and knowledge distillation) is the most relevant direction for narrowing the gap between high-capability models and real-time mobile constraints, enabling more sophisticated architectures to run on-device (87, 88). Where quantization is used quantization-aware training (QAT) should be implemented, where quantization constraints are incorporated during model training rather than applied *post-hoc* (81, 82). This approach significantly reduces accuracy loss compared to post-training quantization and aligns with the “Efficiency with Accuracy Preservation” principle, which emphasizes the importance of maintaining model performance while reducing computational requirements (83, 84).

Several reviewed systems also lacked offline functionality, restricting use in areas without reliable connectivity. To address this, researchers can prioritize on-device processing for all time-critical and safety-related functions, with cloud processing reserved for non-essential enhancements (85, 86). Prioritizing on-device processing for obstacle detection and navigation with cloud processing reserved for non-essential features would also directly address the connectivity limitations identified.

#### 4.2.3 Methodological standardization

The visual prosthesis system showed accuracy ranging from 68.29% to 97.23% across object categories, highlighting generalization challenges when AI models encounter low-resolution and low-contrast conditions. Studies in this review varied substantially in evaluation design, metrics, and reporting, limiting direct comparison and the ability to draw cumulative conclusions across the field. This heterogeneity in evaluation approaches across studies limits both how well current systems’ real-world performance can be judged and how future systems can be benchmarked against them. To enable consistent performance comparison and facilitate meta-analyses, evaluation protocols and reporting practices must be standardized. Structured reporting frameworks similar to CONSORT guidelines adapted for assistive technology would improve study quality and reproducibility (92). At minimum, including shared reference datasets reflecting varied real-world conditions (lighting, weather, crowding) and consistent task protocols for common applications such as navigation and object recognition should be adopted. This will enable future systematic reviews and meta-analyses to compare performance directly rather than narratively.

In all, these findings point to three priorities for future research: robustness, achieved through multi-modal sensor fusion and training on real-world, environmentally diverse datasets; deployability, through model compression and on-device prioritization of safety-critical functions; and evidence quality, through standardized reporting protocols and a shift from pilot studies toward prospective, comparative effectiveness trials with patient-important outcomes.

### 4.3 Limitations of the systematic review

A limitation of this work is that it focused solely on academic studies and published papers. Many AI-based assistive technologies developed or deployed by companies or startups may not be captured in the academic literature, leading to real-world implementations being underrepresented. Additionally, the quality of the included studies could not be systematically assessed because most studies reported system development, experimental validation, or pilot evaluations, rather than formal clinical or controlled trial designs and the studies recorded different classes of metrics (e.g., classification accuracy, AUC, F1 score, localization error in meters, heading error in degrees, and user-rated satisfaction scores on differing scales) reflecting distinct underlying tasks (recognition, navigation, environmental interaction) rather than a single construct that could be converted to a common scale without distortion.

## 5 Conclusion

In conclusion, AI-enabled assistive technologies for low vision rehabilitation have demonstrated technical feasibility and promising performance, yet substantial evidence gaps remain between laboratory capabilities and clinical implementation. While navigation systems approach clinically meaningful accuracy thresholds the environmental limitations prevent definitive conclusions about real-world effectiveness or sustained adoption. Future research must shift from algorithmic innovation toward prospective validation, from proof-of-concept to comparative effectiveness trials, and from surrogate outcomes toward patient-important functional and quality-of-life measures. Future work should prioritize prospective clinical trials evaluating real-world effectiveness, standardized functional outcome measures that enable comparison across studies, and validation on diverse, ecologically representative datasets that capture real-world conditions users encounter outside controlled settings.
