# Supplementary material for: Artificial intelligence innovations in low vision rehabilitation: a systematic review of emerging technologies
Source: Front Digit Health. 2026 Oct 15;8:1896084. doi: 10.3389/fdgth.2026.1896084 (PMC13717276; doi:10.3389/fdgth.2026.1896084)
Supplement: Supplementary file 1 [file Supplementaryfile1.docx]

**Supplementary File 1: Database searches**

1. PubMed

| **Search** | **Query** |
| --- | --- |
| #1 | ("vision, low"[MeSH]) OR "persons with visual disabilities"[MeSH] OR "Visually Impaired Person"[tiab] OR "low vision"[tiab] OR "visual impair*"[tiab] OR "vision loss"[tiab] OR "visually impaired"[tiab] OR "residual vision"[tiab] OR "partial sight*"[tiab]) |
| #2 | ("Artificial Intelligence"[MeSH] OR "Machine Learning"[MeSH] OR "Deep Learning"[MeSH] OR "artificial intelligence"[tiab] OR AI [tiab] OR "machine learning"[tiab] OR "deep learning"[tiab] OR "neural network*"[tiab] OR "computer vision"[tiab] OR ("intelligent system*"[tiab]) |
| #3 | (Rehabilitation [MeSH] OR "self-help devices"[MeSH] OR "Vision Rehabilitation” [ tiab] OR "Assistive Technology"[tiab]) OR rehabilitation[tiab] OR "vision rehabilitation"[tiab] OR "assistive technolog*"[ tiab] OR "adaptive technolog*"[tiab] OR "wearable device*"[tiab] OR "visual aid*"[tiab]) |
| #4 | #1 AND #2 AND #3 |

2. Scopus

| **Search** | **Query** |
| --- | --- |
| #1 | TITLE-ABS-KEY ("low vision" OR "low-vision" OR "visual impairment" OR "visually impaired"OR visual impair* OR "vision loss"OR "residual vision" OR "partial sight*") |
| #2 | TITLE-ABS-KEY ("artificial intelligence" OR AI OR "machine learning" OR "deep learning" OR "neural network*" OR "computer vision" OR "intelligent system*") |
| #3 | TITLE-ABS-KEY ("rehabilitation" OR "vision rehabilitation" OR "assistive technolog*" OR "adaptive technolog*" OR "visual aid*" OR "smart glasses" OR "electronic travel aid*" OR "navigation aid*") |
| #4 | #1 AND #2 AND #3 |

3. Web of Science

| **Search** | **Query** |
| --- | --- |
| #1 | TS**= (**"low vision" OR "low-vision" OR "visual impairment" OR "visually impaired" OR "visual impair*" OR "residual vision" OR "partial sight*" OR "low vision rehabilitation") |
| #2 | TS= ("artificial intelligence" OR AI OR "machine learning" OR "deep learning" OR "neural network*" OR "computer vision" OR "intelligent system*") |
| #3 | TS= ("rehabilitation" OR "vision rehabilitation" OR "assistive technolog*" OR "adaptive technolog*" OR "wearable device*" OR "visual aid*") |
| #4 | #1 AND #2 AND #3 |

4. IEEE Xplore

| **Search** | **Query** |
| --- | --- |
| #1 | ("All Metadata":"rehabilitation" OR "All Metadata":"vision rehabilitation" OR "All Metadata":"assistive technolog*" OR "All Metadata":"adaptive technolog*" OR "All Metadata":"wearable device*" OR "All Metadata":"visual aid*") |
| #2 | ("All Metadata":"artificial intelligence" OR "All Metadata":AI OR "All Metadata":"machine learning" OR "All Metadata":"deep learning" OR "All Metadata":"neural network*" OR "All Metadata":"computer vision" OR "All Metadata":"intelligent system*") |
| #3 | ("All Metadata":"rehabilitation" OR "All Metadata":"vision rehabilitation" OR "All Metadata":"assistive technolog*" OR "All Metadata":"adaptive technolog*" OR "All Metadata":"wearable device*" OR "All Metadata":"visual aid*") |
| #4 | 1# AND #2 AND #3 |
